# Evaluation of Knowledge, Attitudes, and Practices Related to Osteoporosis and Correlates of Perceived High Risk among People Living in Two Main Districts of Lebanon

**DOI:** 10.1155/2022/1188482

**Published:** 2022-05-23

**Authors:** Joanna Nohra, Yonna Sacre, Afif Abdel-Nour, Haider Mannan

**Affiliations:** ^1^Department of Nutrition and Food Sciences, Faculty of Arts and Sciences, Holy Spirit University of Kaslik, P.O Box 446, Jounieh, Lebanon; ^2^School of Engineering, Holy Spirit University of Kaslik, P.O Box 446, Jounieh, Lebanon; ^3^Translational Heath Research Institute, Western Sydney University, Campbelltown, NSW, Australia

## Abstract

**Background:**

The prevalence of osteoporosis is increasing in Lebanon.

**Aim:**

We evaluated the knowledge, attitudes, and practices related to osteoporosis and correlates of its perceived high risk among people living in Beirut and Mount Lebanon districts of Lebanon.

**Methods:**

This study is a cross-sectional study which consisted of 376 participants that were selected from the two districts within two or three households after two geographical areas were randomly selected from each stratum classified by education and altitude. They were then asked to fill a KAP survey on osteoporosis and provide information on factors likely related to its perceived high risk.

**Results:**

The majority of participants had a low (20.2%) and moderate (65.4%) knowledge of osteoporosis, with a higher knowledge in females than in males. A higher percentage of young people perceived it as a serious health risk than that of older people. In contrast, 85.9% participants reported drinking caffeinated beverages and 51.6% participants reported that they do not exercise. Glucose intolerance due to epigenetic and genetic factors, female sex, and older age were risk factors of a perceived high risk of osteoporosis, while any physical exercise, abstention from caffeine for 48 to 72 hours, and higher education were protective factors.

**Conclusion:**

A nationwide KAP study should be conducted; likewise, awareness campaigns should be adopted.

## 1. Introduction

Osteoporosis (OP) is acknowledged as a serious public health problem as it is associated with substantial physical and economic burdens affecting more than 200 million people worldwide [1]. It could also cause serious health issues, such as fractures, which are correlated to a high rate of morbidity and mortality. Fractures contribute to 15–30% of all fatalities, which is comparable to breast cancer and stroke [2, 3]. Hence, there is a need to take precautions for preventing the occurrence of OP. Furthermore, various pharmacological treatments to prevent or treat osteoporosis and minimize the risk of fractures have been identified (hormone replacement therapy, bisphosphonates, calcitonin, and strontium ranelate) [4]. However, some of these treatments may have long-term adverse health outcomes [4].

Every year, millions of people are affected by OP, the majority of whom are women [5]. However, OP is still not given enough attention in the Middle East including Lebanon [6] despite studies showing that this disease is common in this region [7]. The total pooled prevalence rate was reported to be 24.4% in the eastern Mediterranean region based on 31,593 participants [7], and as for Lebanon, the prevalence of OP was assessed to be 33% among women and 22.7% among men in 2018 [3]. The WHO defines OP in women as having a bone mineral density (BMD) of at least 2.5 standard deviations below the mean of young, healthy women evaluated by dual-energy *X*-ray absorptiometry (DEXA) in the hip or spine [8]. Two types of osteoporosis can be identified: primary and secondary [9]. Primary osteoporosis is the most frequent type and is subdivided into two types: type I that develops in postmenopausal women as a result of estrogen shortage and type II caused by the natural aging process [10]. Secondary osteoporosis, on the other hand, is caused by bone loss induced by specific medical conditions [9]. Also, this disease is more common among the elderly, indicating that it has public health importance in terms of disease burden as, by the year 2050, 40% of the Lebanese population is projected to be 50 years and older [3]. In addition, this disease is the result of mostly modifiable factors (vitamin D status, caffeine and alcohol use, calcium levels, exercise, and smoking) and a few nonmodifiable factors (age, heredity, and gender) [2–6]. It means that this disease is mostly preventable.

While there has been a study [2] to investigate knowledge of OP in Lebanon and its predictors, there has been no study to investigate attitudes and practices of people regarding OP and what relates to taking preventive measures to decrease the perceived risks of developing OP in terms of identifying the risk factors. The importance of this study is illustrated by the fact that the incidence of OP is increasing in the Middle East [11], including Lebanon [12]. Therefore, shedding light on this subject can be judged important in order to increase people's awareness of the disease and help them detect it early or prevent it.

Accordingly, the aim of this study is to assess the KAP of people living in two districts of Lebanon regarding OP and which factors relate to the perceived high risk of OP. Ultimately, this study aims at issuing practical and context-specific recommendations to limit or prevent the risk of developing OP.

## 2. Materials and Methods

### 2.1. Study Design and Recruitment

Our study was cross-sectional and conducted in two major districts in Lebanon, targeting healthy adults aged above 20 years. The number of participants was calculated based on the formula of Krejcie and Morgan (1970) cited by Goyette (2015) [13], given as follows:

(1) $$\begin{array}{c} n=\frac{X^{2}NP\left(1-P\right)}{d^{2}\left(N-1\right)+X^{2}P\left(1-P\right)}, \end{array}$$

where *n* = sample size, *X*^2^ = the table value of the chi-squared statistic for one degree of freedom at the desired 5% level of significance = 3.841, *N* = the population size, *P* = the population proportion (0.5), and *d* = the degree of significance (0.05).

This gave us a sample size of 315. Assuming 20% nonresponse, we increased the sample size to 394, of which 376 participated in the study. There was an 80 : 20 sample split in adults between Mount Lebanon and Beirut districts based on a representative Demographic and Social Statistics Survey (DSSS) by the Central Administration of Statistics (CAS), Lebanon [14]. So, the sample sizes for the two districts in our study were found proportionately.

The data collection started in June 2019 and ended in August 2019. After choosing the regions (Beirut and Mount Lebanon) to conduct the study, we adopted stratified cluster sampling to select the study participants. We stratified Beirut and Mount Lebanon on the basis of altitude of the area and the sociodemographics of the population living there. Specifically, the sociodemographic factor which was considered to differentiate between the strata was the education level as knowledge of OP might be generally low among less educated than more educated. Only a small part of Mount Lebanon is like Beirut in terms of altitude (being at sea level), while the majority has higher altitude (being at above sea level) than Beirut.

**Figure 1:**
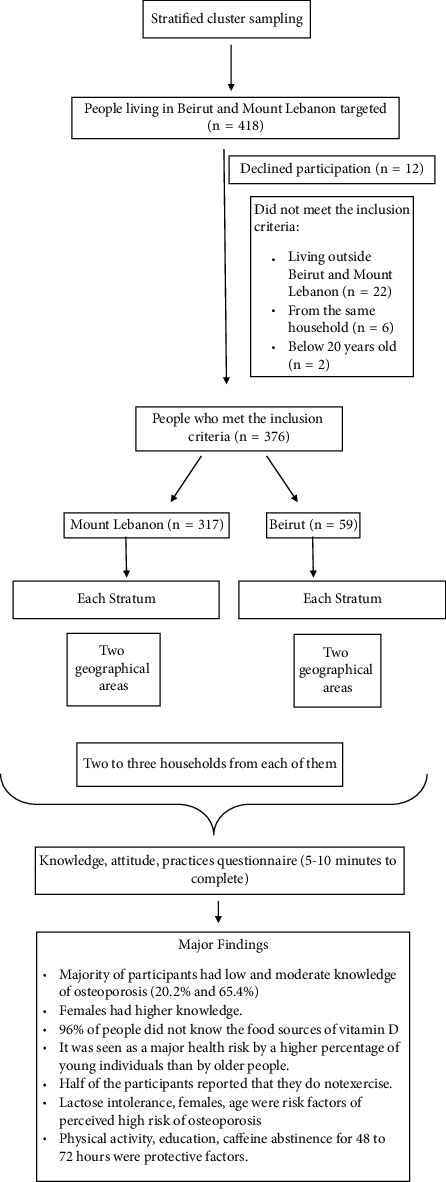
Study plan.

Subsequently, we chose two geographic areas randomly from each stratum and then randomly selected two to three households from each of them. Only one person from the same household who was either a male or a female aged over 20 years was requested to participate in the survey. A study plan is shown in Figure 1.

### 2.2. Data Collection Instrument: KAP Survey

The data were collected using a questionnaire consisting of closed and open questions. It was developed and conducted to note the KAP of people in what is related to OP and its risk factors. It was developed in English and translated to Arabic based on a thorough literature review and review of internationally used questionnaires [15–17]. Some of the questions like the qualitative questions were validated having good content validity based on a pilot study of 20 Lebanese adults living outside the area of the study. Its main sections were as follows: The Socio-demographics section covers questions related to the demographic characteristics of participants such as their age, gender, education level, and place of residence. The Knowledge section covers questions related to the knowledge of participants in what relates to osteoporosis and its risk factors. The Attitude section covers questions related to the attitude of participants in what relates to knowing more about osteoporosis and its risk factors, taking preventive measures, and consulting specialists.

The Practice section covers questions related to the current practices of participants in what relates to osteoporosis and its risk factors. The purpose of the study and the time needed to fill out the questionnaire were communicated to the participants before starting the survey.

To measure the knowledge level, the Knowledge section has been coded and a general knowledge score was given to each participant. To be able to do so, all the correct answers have been given a score of three, while a score of 2 has been given to the answers, half of which have been correct, and a score of 1 has been given to the wrong answers. After that, the results were distributed into three categories (low, moderate, and high). The highest score “63” means that the person has answered all 21 questions correctly, while a score of “21” means that the person has answered all the questions wrong. In order to obtain the number of intervals which is 14, the researcher multiplied the number of questions by the highest note which is 3 as well as by the lowest note which is 1. Then, the researcher subtracted these two numbers, and we divided them by 3. Accordingly, people with a score varying from 21 to 35 were considered as having a low knowledge. Likewise, a person with a score varying from 36 to 49 was classified as having a moderate knowledge. In addition, those with a score varying from 50 to 63 were considered to have a high knowledge.

### 2.3. Ethics

Ethical approval to conduct the study was obtained from the Ethical Committee of the Holy Spirit University of Kaslik. Moreover, an informed consent form was provided to all participants so that they can withdraw from the study at any time. This study did not result in any physical or psychological harm to the participants. On the other hand, the credentials of the participants were kept confidential, and the results of the study were anonymous and used only for academic purposes.

### 2.4. Data Analysis

Basic descriptive statistics were used to present and describe all the findings of the KAP study. These included percentages and frequencies. In addition, for categorical variables such as age groups and gender, the Rao–Scott test was used to assess the association of each with the KAP variable. For the multivariate analysis to determine the significant predictors of perceived high risk of OP, we used survey logistic regression to account for the complex survey design and adjust for oversampling of females in the sample. The predictors included were sex, age, education level, any physical exercise including multimodal training, exemption from caffeine for 48 to 72 hours, and lactose intolerance due to epigenetic and genetic factors. All data analyses were performed using SAS version 9.4 [18].

## 3. Results

### 3.1. Description of the Participants' Characteristics

The study population constituted 376 persons who consented to participate within the KAP survey. The participants were predominantly (84.3%) from Mount Lebanon, 50.64% were males, 54.5% were between 20 and 30 years while 18.5% were between 31 and 40 years, and 77.1% had university education indicating that the participants were mostly educated as well as young. The results are shown in Table 1.

### 3.2. Evaluation of the Knowledge Level of the Study Population

**Figure 2:**
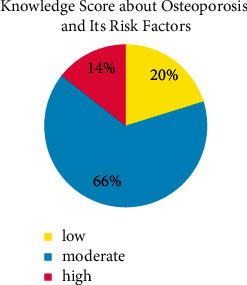
Knowledge score about osteoporosis and its risk factors.

The distribution of the level of knowledge about OP is shown in Figure 2. Accordingly, most participants (65.4%) had a moderate knowledge about OP, followed by low (20.2%) and high (14.4%).

**Figure 3:**
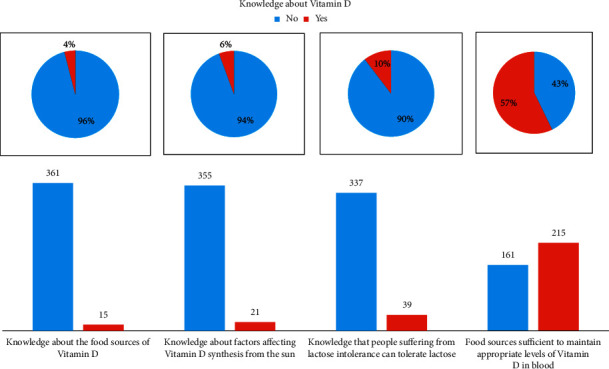
Knowledge about vitamin D.

The majority (*n* = 361 or 96%) of 376 participants did not know the food sources of vitamin D. Moreover, 355 (94.4%) participants did not know the factors affecting the vitamin D synthesis from the sun. Furthermore, 337 (89.6%) participants did not know that people suffering from lactose intolerance can also tolerate a certain amount of lactose. These results are presented in Figure 3.

**Figure 4:**
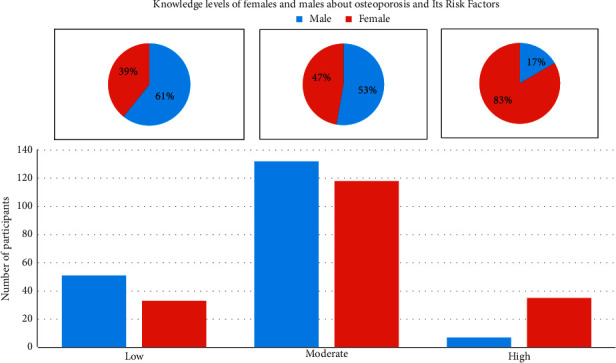
Knowledge levels of females and males about osteoporosis and its risk factors.

Furthermore, females had a higher knowledge of OP than male participants (see Figure 4). In fact, of all women, 33 (17.7%) had a low knowledge level, 118 (63.4%) had a moderate level of knowledge, and 35 (18.8%) had a high level of knowledge compared to 51 (26.8%) with a low knowledge level, 132 (69.5%) with moderate knowledge, and only 7 (3.7%) with a high knowledge level among men. These percentages were significantly (*p* value ≤0.001) different between men and women.

In addition, the results of cross tabulation showed that participants under the age of 40 had a significantly (*p* value ≤0.01) higher level of knowledge in what relates to OP and its risk factors (results not shown).

### 3.3. Assessment of Participants' Attitudes

Remarkably, a large majority (89.4%) of the people perceived OP as a serious health risk. A higher percentage of young people (*p* value ≤0.05) from 20 to 30 years perceived OP as a serious health risk than that of older people. When asked if they would like to know more about this topic, 259 (68.9%) participants replied in the affirmative. Participants further explained that they would like to know more about the topic through conferences, articles, books, awareness campaigns, dieticians, the Internet, social media, and the general media.

### 3.4. Evaluation of the Practices Related to OP

Regarding the practices of people in what relates to taking food supplementation and sun exposure, 111 (29.5%) participants take food supplements while 215 people (57.2%) seek the direct sun. In what relates to caffeine consumption, 323 (85.9%) participants drink caffeinated beverages. Yet, 210 (55.9%) participants can go 48 to 72 hours without caffeine. In addition, 194 (51.6%) participants do not exercise. The rest of the people (48.4%) go to the gym, conduct regular exercise, attend gym classes, and perform yoga, Pilates, jogging, and various other physical activities.

### 3.5. Correlates of Perceived High Risk of OP

We found that those who perform any physical exercise have significantly (*p* ≤ 0.0001) lower odds (37.8%) of perceived high risk of OP. Those who abstain from caffeine for 48 to 72 hours had significantly (*p* ≤ 0.0001) lower odds (37.2%) of perceived high risk of OP. Females had significantly (*p* ≤ 0.0001) higher odds (56.5%) of perceived high risk of OP. Those who had lactose intolerance due to epigenetic and genetic factors had significantly (*p* ≤ 0.0001) higher odds (9.7%) of perceived high risk of OP. There was a significant (*p* ≤ 0.0001) positive relationship between the age and perceived high risk of OP with the odds increasing (15.2%) for every single unit increase in age. There was a significant (*p* ≤ 0.0001) negative relationship between the education level and perceived high risk of OP with the odds decreasing with an increase in the educational level. The results are summarized in Table 2.

## 4. Discussion

It is important to highlight that 83.8% or 315 participants reported having heard about OP, which is in accordance with another study in Saudi Arabia [19]. Furthermore, the knowledge difference between females and males was significant for some questions and not significant for others. Also, the findings of another study in Malaysia [20] were consistent with ours where they found that women have higher knowledge than men do.

A very high percentage of the participants did not know about the food sources of vitamin D (*n* = 361, 96%). This is consistent with studies in Pakistan where the studied group had little knowledge regarding the food sources of vitamin D [21]. Also, this might affect the ability of people to eat food high in vitamin D as a study showed that people who are aware of vitamin D food sources consume more of these products [22]. Moreover, a study showed that vitamin D-deficient patients had less knowledge about the sources of vitamin D compared to those who were healthy [23].

In addition, 94.4% or 355 participants were unaware of the elements that influence vitamin D production from sunlight. Many factors can influence this formation, including exposure period, cloud cover, skin melanin concentration, and sunscreen [24]. In general, if people are unaware of these aspects and act counter to recommendations, this can result in a decline in synthesis. We were not able to examine the impact of the factors affecting the vitamin D synthesis from the sun due to lack of information. It can be noted that most of the vitamin D in any population comes from sun exposure, since only a small number of food items contain vitamin D [25].

Most of the participants (*n* = 337, 89.6%) did not know that people suffering from lactose intolerance can actually tolerate a certain amount of lactose. Thus, people with lactose intolerance may tend to avoid all products containing lactose, which will result in a decrease in calcium intake. In other studies, people with lactose intolerance were found to tolerate up to 12 grams of lactose [26] and even 18 grams when paired with other foods [27].

The results of the attitude section indicate that the majority of participants perceive OP as a serious health risk and are willing to consult specialists (like physicians and dieticians) if they are at high risk of developing it. This finding is in accordance with another study in which a similar attitude was observed [28].

We found lactose intolerance due to epigenetic and genetic factors, female sex, and older age to be risk factors of perceived high risk of OP, while any physical exercise, abstention from caffeine for 48 to 72 hours, and higher education level were found to be protective factors of perceived high risk of OP. Any physical exercise includes going to the gym, conducting regular exercise, attending gym classes, and performing yoga, Pilates, jogging, and other physical activities including sports. This finding is consistent with another study, which revealed that a higher education level was associated with a higher level of awareness [29].

As for practices related to reducing the risk of OP, 215 or 57.2% people reported seeking the direct sun. However, this conclusion may be insufficient in terms of sun exposure because it does not account for factors that influence vitamin D production from the sun, such as the use of sunscreen, the length of time spent in the sun, and the amount of time spent outdoors. Similarly, traditions can influence this synthesis; for example, in Lebanon, veiled women had a much greater rate of vitamin D insufficiency [30].

Furthermore, 194 (51.6%) participants reported that they do not exercise. However, exercise was shown to be a preventive factor in the development of OP in a study [31], and another study focused on the adequate type of sport to prevent OP and falls, where multimodal exercise training, which consists of weight-bearing exercises, progressive resistance training, and balanced mobility training, is recommended [32].

This study has several strengths and few limitations. First, we used a probabilistic sample design to collect our data which enabled us to get a sample which was demographically representative of the target population of Beirut and Mount Lebanon combined. Also, this is the first KAP study on OP ever conducted in Lebanon. However, it was conducted in only two districts of Lebanon and so cannot be considered to be representative of the entire population of Lebanon. There are some lessons to be learnt from this study which might help better conduct a similar study in Lebanon or a similar population. In regards to vitamin D from sun exposure, we did not collect data on the factors affecting the vitamin D synthesis from the sun. For physical exercise, we were not able to identify the average time spent on training and whether people were performing multimodal exercises. As for calcium intake, we did not collect information regarding this notion.

## 5. Conclusions and Recommendations

The present study suggests that more attention should be paid to OP in Lebanon. In fact, our findings urge the need for educating the Lebanese population in general, and that of Beirut and Mount Lebanon in particular, about this critical disease. The perceived risk associated with this disease is higher among the less educated and those regularly consuming caffeinated drinks. Also, the perceived risk associated with this disease is higher among those not performing any physical exercise. These are all modifiable risk factors. We hope that this study will pave the way for further studies having larger sample sizes and for the entire population of Lebanon. Such studies will indicate whether the results we found for Beirut and Mount Lebanon are generalizable to the entire population of Lebanon. This can be achieved by targeting all Lebanese regions and involving community parties and associations to help recruit participants from the entire population.

Based on the results of this study, the following practical recommendations are presented:

- Conducting a nationwide KAP study about OP is very important to have a better understanding of this debilitating disease among Lebanese adults and the ability to conduct appropriate activities.
- Knowing that this study showed a certain gap in the knowledge section and conducting more awareness campaigns on this topic would help provide people with more accurate and complete information. People of lower education or those of a lower socioeconomic status should be targeted to increase awareness of this disease. People should be made aware of the factors affecting the vitamin D synthesis from the sun, eating vitamin D-enriched diet, and not consuming caffeinated drinks on regular basis. Also, different types of physical exercise including multimodal exercise classes can be promoted in the two districts. Since the awareness was very low regarding being able to eat a certain amount of lactose for those having the condition of lactose intolerance, this may indicate that lots of people with this health condition may have been avoiding dairy products and thereby reducing their calcium intake. This explanation may be supported by our finding that those having lactose intolerance had significantly higher odds of a perceived high risk of developing OP.
- The general awareness of OP and the promotional activities discussed above can be provided by people through dieticians, advertisements on social media, or newspapers, radio, and television. Also, this can be performed through collaboration with family and general physicians who have a major role in guiding people to preventive medicine.
- Adopting a multidisciplinary approach consisting of the distribution of work between different health professionals is proposed for better outcomes.

## Figures and Tables

**Table 1:** Sample characteristics (sociodemographic factors and anthropometric measures).

Variables	Case (patients) (*N* = 376)	
	Number	Percentage
Age		
20 to 30 years	205	54.5
31 to 40 years	62	18.5
41 to 50 years	33	8.8
50 to 60 years	36	9.6
> or equal to 60 years	40	10.6
Sex		
Male	190	50.64
Female	186	49.36
Educational status		
No school	8	2.1
Primary school	21	5.6
Secondary school	57	15.2
Undergraduate	106	28.2
Postgraduate	184	48.9
Location		
Mount Lebanon	317	84.3
Beirut	59	15.7

**Table 2:** Estimated odds ratios, 95% confidence intervals (CIs), and the Wald Z statistic for the effects of selected variables on odds of perceived high risk of osteoporosis in Lebanon based on survey logistic regression.

Covariate	Odds ratio	95% CI
Physical exercise		
Yes vs. no	0.632^*∗*^	0.604–0.662
Caffeine abstention for two to three days		
Yes vs. no	0.628^*∗*^	0.600–0.658
Lactose intolerance due to epigenetic and genetic factors		
Yes vs. no	1.097^*∗*^	1.046–1.150
Gender		
Female vs. male	1.565^*∗*^	1.494–1.639
Age	1.152^*∗*^	1.130–1.175
Education level		
Secondary vs. none/elementary	0.434^*∗∗*^	0.395–0.477
College vs. none/elementary	0.340^*∗*^	0.310–0.370
University vs. none/elementary	0.336^*∗*^	0.305–0.371

The statistical tests and 95% confidence intervals are both based on the Wald method. ^*∗*^*p* ≤ 0.0001 and ^*∗∗*^*p* ≤ 0.001.

## Data Availability

The datasets used and/or analysed during the current study are available from the corresponding author on reasonable request.

## Abbreviations

CAS: Central Administration of Statistics
DEXA: Dual-energy X-ray absorptiometry
DSSS: Demographic and social statistics survey
KAP: Knowledge, attitude, and practice
OP: Osteoporosis.
